# High mitochondrial DNA levels accelerate lung adenocarcinoma progression

**DOI:** 10.1126/sciadv.adp3481

**Published:** 2024-11-01

**Authors:** Mara Mennuni, Stephen E. Wilkie, Pauline Michon, David Alsina, Roberta Filograna, Markus Lindberg, David E. Sanin, Florian Rosenberger, Alina Schaaf, Erik Larsson, Erika L. Pearce, Nils-Göran Larsson

**Affiliations:** ^1^ Division of Molecular Metabolism, Department of Medical Biochemistry and Biophysics, Karolinska Institutet, Stockholm, Sweden.; ^2^ Department of Medical Biochemistry and Cell Biology, Institute of Biomedicine, Sahlgrenska Academy at University of Gothenburg, Gothenburg, Sweden.; ^3^ Bloomberg-Kimmel Institute of Immunotherapy, Department of Oncology, Johns Hopkins University School of Medicine, Baltimore, MD, USA.; ^4^ Max Planck Institute of Biochemistry, Department of Proteomics and Signal Transduction, Munich, Germany.

## Abstract

Lung adenocarcinoma is a common aggressive cancer and a leading cause of mortality worldwide. Here, we report an important in vivo role for mitochondrial DNA (mtDNA) copy number during lung adenocarcinoma progression in the mouse. We found that lung tumors induced by KRAS^G12D^ expression have increased mtDNA levels and enhanced mitochondrial respiration. To experimentally assess a possible causative role in tumor progression, we induced lung cancer in transgenic mice with a general increase in mtDNA copy number and found that they developed a larger tumor burden, whereas mtDNA depletion in tumor cells reduced tumor growth. Immune cell populations in the lung and cytokine levels in plasma were not affected by increased mtDNA levels. Analyses of large cancer databases indicate that mtDNA copy number is also important in human lung cancer. Our study thus reports experimental evidence for a tumor-intrinsic causative role for mtDNA in lung cancer progression, which could be exploited for development of future cancer therapies.

## Teaser

High mtDNA levels increase tumor burden in mice through enhanced mitochondrial respiratory chain capacity.

## INTRODUCTION

Warburg’s influential work in the early 1920s highlighted tumor cells’ preference for glycolysis even in the presence of oxygen, the so-called “Warburg effect,” suggesting impaired mitochondrial respiration to be the driving force behind tumorigenesis (*1*). In the past decades, mitochondria, traditionally merely considered the cellular powerhouses, have re-emerged as key players in cancer biology (*2*). Mitochondria are the metabolic hubs of the cell, and it is now established that they are essential for cancer progression, as they provide metabolites necessary for cell proliferation and support the metabolic plasticity needed to adapt to the changing tumor microenvironment (*3*–*7*). Despite the reignited interest, numerous questions regarding the exact mechanisms linking mitochondria to cancer initiation and progression remain. The role of mitochondria in tumor development seems to be highly organ dependent and varies with cancer type and stage (*4*, *8*–*10*).

The mitochondrial DNA (mtDNA) is present in thousands of copies in the cell, and mtDNA levels typically correlate with the cellular metabolic needs. The mitochondrial transcription factor A (TFAM) packages mtDNA into nucleoids and plays a critical role in mtDNA maintenance and expression (*11*). TFAM modulates mtDNA levels, thus influencing mitochondrial function (*12*, *13*). The amount of mtDNA is known to vary greatly during tumor progression, although the direction of the change varies in different cancer types (*14*–*16*).

Lung cancer remains the deadliest cancer among men in Europe (*17*), with non–small cell lung cancer (NSCLC) representing the most prevalent subtype. Among NSCLC, lung adenocarcinoma (LUAD) is the most common type of lesion (*18*, *19*), and it arises from mutations in the *KRAS* gene in 30% of the cases (*20*). Despite advances in diagnosis and treatment, the overall prognosis for patients with KRAS-driven lung cancer remains poor, underscoring the urgent need for a deeper understanding of the underlying molecular mechanisms.

Recent studies in mice have provided evidence that LUAD is highly dependent on mitochondrial respiration (*21*, *22*). Moreover, analyses of large datasets from patients with cancer have revealed that lung cancer exhibits high mtDNA levels compared to normal lung tissue (*14*, *15*). These results highlighted the potential importance of mitochondrial function and mtDNA level variation during LUAD development.

Here, we experimentally studied the connection between lung cancer and mtDNA levels in vivo by using a variety of mouse mutants in which an inducible expression of a mutant *Kras^G12D^* (*23*, *24*) allele was combined with genetic alterations that cause reduced (*12*) or increased (*25*) mtDNA copy number. We found that tumors isolated from *Kras*^G12D^ mice contained higher mtDNA copy number and oxidative phosphorylation (OXPHOS) protein levels compared with the surrounding nonaffected tissue. We extensively characterized mitochondrial function and found that KRAS-driven lung tumors have high respiratory capacity. Genetically increasing mtDNA copy number before activating the expression of the oncogenic KRAS^G12D^ in the lung strongly enhanced tumor burden in mice, whereas mtDNA depletion in tumor cells diminished tumor volume. We conclude that mtDNA is a key player in LUAD development and report that high mtDNA levels confer a functional advantage to cancer cells, resulting in increased tumor burden in vivo.

## RESULTS

### High mtDNA levels correlate with enhanced expression of mitochondrial respiratory genes in LUAD

**Fig. 1.**
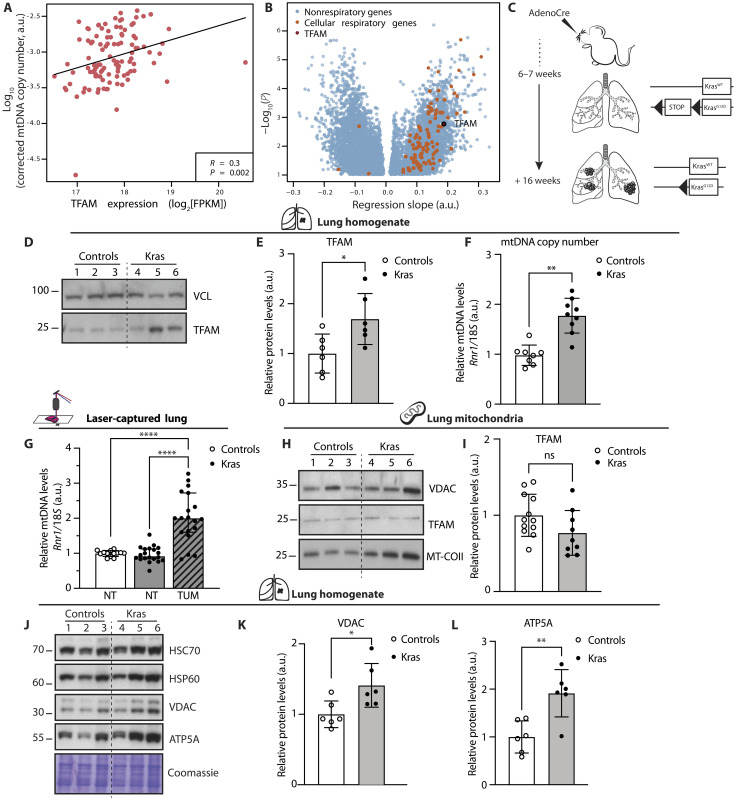
The levels of mtDNA are increased in human and mouse LUAD. (**A**) Correlation coefficient and *P* value for the regression slope between *TFAM* expression and mtDNA levels in LUAD (*14*). (**B**) Linear regression models fitted with gene expression as explanatory and mtDNA levels as response variables. FPKM, fragments per kilobase per million reads. (**C**) Schematic timeline from tumor induction to collection of the *Kras*^G12D^ model. (**D**) Western blot analyses showing the steady-state levels of TFAM in lung homogenates from controls and Kras mice; vinculin (VCL) is used as loading control. (**E**) Densitometric quantification of TFAM protein levels from Western blots. *N* = 6; *P = 0.0250. (**F**) Relative mtDNA levels measured in lung homogenate from controls and Kras mice. *N* > 8; **P = 0.0058. (**G**) mtDNA levels measured in laser-captured tumor portions (TUM) and normal lung (NT). Controls: *N* = 14, Kras: *N* = 20 ****P < 0.0001. (**H**) Western blot analyses of TFAM and cytochrome *c* oxidase subunit 2 (MT-COII) levels in lung mitochondria; voltage-dependent anion channel (VDAC) is used as loading reference. (**I**) Quantification of TFAM protein levels from Western blots. Controls: *N* = 12; Kras: *N* = 9; ns, not significant. (**J**) Western blot analyses of the steady-state levels of heat shock protein 60 (HSP60), VDAC, and adenosine 5′-triphosphate (ATP) synthase F1 subunit alpha (ATP5A) in lungs; heat shock cognate 70 (HSC70) and Coomassie are used as loading references. (**K** and **L**) Quantifications of VDAC and ATP5A levels from Western blots normalized against the loading control. *N* = 6; *P = 0.0198 and **P = 0.0038. Scatter plots show individual data points and the mean values ± SD. Two-tailed *t* test was used for statistical analysis of (E), (F), (I), (K), and (L), and one-way analysis of variance (ANOVA) was used for (G). a.u., arbitrary unit.

Previous analyses of large datasets of patients with cancer revealed that lung cancer is characterized by high mtDNA levels compared to normal lung tissue (*14*, *15*). Moreover, recent work on mouse models reported that LUAD is highly dependent on mitochondrial respiration (*21*, *22*). Therefore, we hypothesized that the levels of mtDNA could be important for tumorigenesis in the lung. Because TFAM protein levels directly control mtDNA copy number (*13*, *25*), we assessed both mtDNA and *TFAM* transcript levels in LUADs from patients. To this end, we analyzed data deposited in The Cancer Genome Atlas (TCGA) database and found a positive correlation between mtDNA copy number and *TFAM* mRNA expression in LUAD (*n* = 107) (Fig. 1A). The expression of cellular respiration-annotated genes was enriched among genes that positively correlated with mtDNA levels (Fig. 1B). These data show that mtDNA copy number is accompanied by increased *TFAM* transcript levels and that higher mtDNA levels are associated with changes in gene expression that sustain mitochondrial respiration in human LUAD samples.

### The levels of mtDNA are increased upon KRAS-driven transformation in lung cancer in mice

To investigate the role of mtDNA levels during lung tumor progression in vivo, we used a well-established inducible KRAS^G12D^-driven lung cancer mouse model. The expression of the mutant oncogenic KRAS^G12D^ protein was activated by nasal delivery of adenoCre, leading to tumor development in the lungs (Fig. 1C and fig. S1, A and B) (*23*, *24*). We found a significant increase in TFAM protein (Fig. 1, D and E) and mtDNA (Fig. 1F) levels in lysates from lung tissue isolated from *Kras*^G12D^ mice (hereafter referred to as Kras mice) in comparison with wild-type mice treated with adenoCre (Fig. 1, D to F). The increase in mtDNA levels was more significant when the mtDNA content was measured in DNA extracted from laser-captured tumor portions and compared with normal lung tissue from wild-type animals or normal lung tissue from animals with tumors (Fig. 1G). These findings show that mtDNA copy number is specifically increased in tumor tissue upon activation of KRAS^G12D^ expression. No difference in TFAM transcript levels between Kras and wild-type animals was observed (fig. S1C), in line with evidence from our previous work showing that regulation of mitochondrial proteins mostly occurs at the posttranscriptional level in the mouse (*26*, *27*). We proceeded to analyze the TFAM protein content in mitochondria isolated from lung tissue and found no difference between controls and Kras samples (Fig. 1, H and I). We hypothesized that an increase in mitochondrial content per cell could explain this finding. To test this possibility, we assessed levels of the voltage-dependent anion channel (VDAC) and the heat shock protein 60 (HSP60), which are established markers for mitochondrial mass, in protein extracts from lung homogenates of Kras and control mice (Fig. 1J). We found a mild but significant increase of mitochondrial content in Kras samples (Fig. 1, J and K). Moreover, we observed a pronounced enrichment for the mitochondrial adenosine 5′-triphosphate (ATP) synthase subunit alpha (ATP5A) in Kras samples compared to controls (Fig. 1, J and L). Notably, the steady-state levels of cytochrome *c* oxidase (COX) subunit 2 (MT-COII) were also higher in mitochondria isolated from Kras lung tissue than controls (Fig. 1H), suggesting that a further enhancement of OXPHOS subunits per mitochondrion occurs in KRAS-driven LUAD. Together, these data show a spontaneous up-regulation of mtDNA copy number, mitochondrial mass, and mitochondrial OXPHOS protein levels in KRAS-driven LUAD in the mouse, mirroring to some extent what occurs in human LUAD samples (Fig. 1, A and B). These findings argue that the mouse model used recapitulates important features of human LUAD.

### Increasing mtDNA levels before tumor development enhances tumor burden

**Fig. 2.**
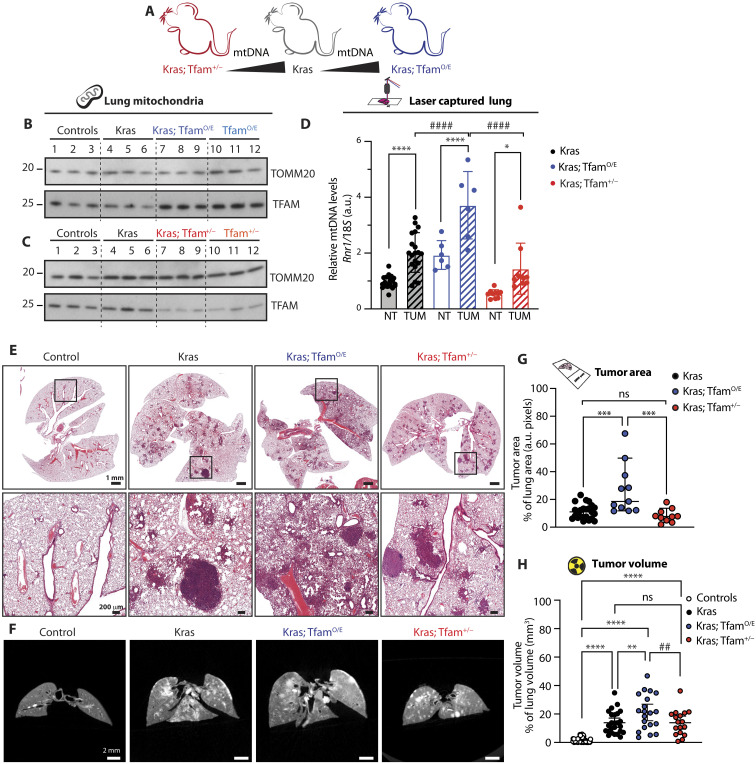
Increasing mtDNA levels before tumor induction increases tumor burden in mice. (**A**) Schematic representation of the mouse models used to generate Kras-driven LUADs with different mtDNA levels. (**B** and **C**) Representative Western blot analyses of TFAM steady-state levels in mitochondria isolated from lung of controls, Kras, Tfam^O/E^, Kras; Tfam^O/E^ (B), Tfam^+/−^, and Kras; Tfam^+/−^ (C). Mitochondrial import receptor subunit TOM20 (TOMM20) is used as a loading control. (**D**) Relative mtDNA levels measured in laser captured tumor portions (TUM) and normal surrounding lung tissue (NT) isolated from the aforementioned groups. Scatter plots show individual data points and the mean values ± SD. One-way ANOVA was used for the statistical analysis; Kras: *N* = 20, Kras; Tfam^O/E^: *N* = 6, and Kras; Tfam^+/−^: *N* = 10. *P = 0.0185, ****P < 0.0001, and ####*P* < 0.0001. (**E** and **F**) Representative hematoxylin and eosin (H&E) staining of lung tissue sections (E) and μCT images (F) of the lungs from controls, Kras, Kras; Tfam^O/E^, and Kras; Tfam^+/−^ mice. Scale bars, H&E: 1 mm (top) and 200 μm (bottom) and μCT: 2 mm. (**G**) Tumor area over total lung area quantified from H&E sections. Scatter plots show individual data points and the median + 95% confidence interval (CI) for each group. Statistical significance was calculated with one-way ANOVA test. Kras: *N* = 23; Kras; Tfam^O/E^: *N* = 11, and Kras; Tfam^+/−^: *N* = 10; ***P = 0.0004. (**H**) Quantification of tumor volume and airways from μCT 3D images expressed as percentage of total lung volume. Scatter plots show individual data points and the median + 95% CI. Statistical significance was calculated with one-way ANOVA test. Controls: *N* > 30; Kras: *N* = 25; Kras; Tfam^O/E^: *N* = 20; and Kras; Tfam^+/−^: *N* = 17. ****P < 0.0001, **P = 0.032, and ##*P* = 0.0092.

To understand the cause-and-effect link between the increase in mtDNA copy number and lung cancer development, we took advantage of well-characterized mouse models with different levels of mtDNA due to altered expression of TFAM. We generated mouse lines carrying the inducible *Kras^G12D^* allele in combination with increased (*25*, *28*) or decreased *Tfam* expression (*12*) (Fig. 2A). The mouse with moderate TFAM overexpression has been generated in our laboratory and is heterozygous for a bacterial artificial chromosome (BAC) transgene containing the *Tfam* gene (genotype *Tfam*^+/BACtg^, hereafter denoted Tfam^O/E^). This mouse model presents a ~50 to 100% increase of mtDNA levels in all tissues tested (*25*, *28*). Similarly, heterozygous *Tfam* knockout mice (hereafter, Tfam^+/−^ mice) were generated previously and have ~50% decrease of mtDNA copy number in all tissues tested (*12*, *29*). The changes in TFAM protein levels in these models are mild and affect mtDNA levels without having any major impact on mtDNA gene expression and OXPHOS function (*12*, *25*, *29*).

We assessed TFAM protein levels from lung of Tfam^O/E^ and Tfam^+/−^ mice, with or without the activation of the mutant *Kras* allele, and found changes in TFAM protein levels consistent with the genotypes (Fig. 2, B and C). TFAM steady-state levels in isolated lung mitochondria were increased in Tfam^O/E^ and decreased in Tfam^+/−^ mice (Fig. 2, B and C). We measured the levels of mtDNA in lung homogenate from wild-type, Tfam^O/E^, and Tfam^+/−^ mice and found that the changes in mtDNA levels followed the *Tfam* gene dosage (fig. S1D). Next, we measured mtDNA copy number in laser-captured lung samples from Kras, Kras; Tfam^O/E^, and Kras; Tfam^+/−^ mice and found a consistent increase in mtDNA copy number in captured tumor tissue compared with normal surrounding lung tissue (Fig. 2D). The KRAS-driven increase in mtDNA copy number occurred in all groups, but the degree of the increase was proportional to the setpoint of mtDNA levels determined by the *Tfam* gene dosage (Fig. 2D). To assess whether the preexisting mtDNA levels present before the induction of tumors would affect tumor size, we measured the tumor area on hematoxylin and eosin (H&E)–stained lung sections (Fig. 2E and fig. S1, E and F) and tumor volume on dissected whole lungs by micro-computed tomography (μCT) (Fig. 2F and movies S1 to S20). With both methods, we found that mice with higher mtDNA copy number (Kras; Tfam^O/E^) presented increased tumor burden in comparison with Kras mice (Fig. 2, G and H). In contrast, no significant difference in tumor burden was identified between Kras and Kras; Tfam^+/−^ mice (Fig. 2, G and H), showing that ~50% decrease in mtDNA levels is not limiting the tumor progression in the lung. The enhanced tumor burden of Kras; Tfam^O/E^ mice was not mediated by the presence of an unrelated gene on the BAC clone used to overexpress TFAM because overexpressing TFAM in the Tfam^+/−^ background (genotype: Kras; Tfam^+/−^; Tfam^O/E^) resulted in a tumor burden comparable to the burden in Kras mice (fig. S1G). These results demonstrate that an increase of mtDNA copy number before tumor induction is sufficient to increase the lung tumor burden in mice.

A link between reactive oxygen species (ROS) formation by the OXPHOS system and tumor formation has been previously reported in Kras-driven lung cancer models (*30*), and we therefore assessed mitochondrial aconitase 2 (ACO2) activity, an established marker for damage induced by high levels of superoxide (*31*, *32*). We found no evidence of increased superoxide in mitochondria isolated from lungs of Kras mice with or without changes in TFAM levels (fig. S1H). In contrast, we observed enhanced ACO2 activity and steady-state levels in all models bearing the oncogenic *Kras*, regardless of mtDNA content (fig. S1, I and J). This mean that superoxide-induced damage is likely not a major player in our models, at least not at the experimental time point chosen. The increase in ACO2 activity and levels could reflect a general increase of metabolic activity of tumor cells.

### Immune cell populations in the lung and cytokine levels in plasma are not affected by increased mtDNA levels

**Fig. 3.**
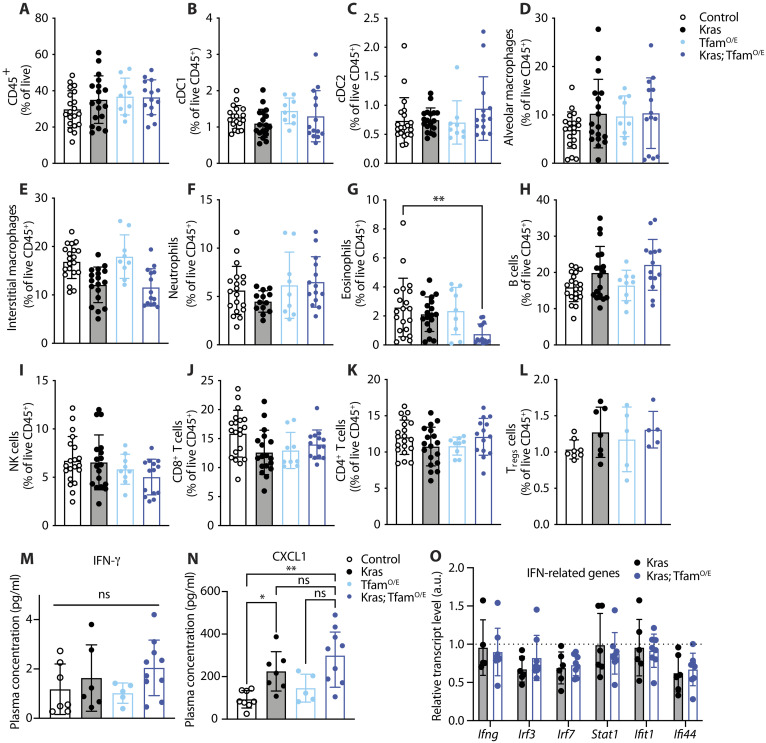
The increase in mtDNA levels does not influence the immune cell populations in the lung and cytokine levels in plasma. (**A** to **L**) Relative quantification of immune cells populations analyzed in lungs from control, Kras, Tfam^O/E^, and Kras; Tfam^O/E^ mice. (A) Total immune cells (CD45^+^). (B and C) Dendritic cells type 1 and 2 (cDC1 and cDC2). (D and E) Alveolar and interstitial macrophages. (F) Neutrophils. (G) Eosinophils. (H) B cells. (I) Natural killer (NK) cells. (J and K) CD8^+^ and CD4^+^ cells. (L) T regulatory cells (T_regs_). **P = 0.005. (**M** and **N**) Scatter plots of the levels of selected cytokines and chemokines: interferon-γ (IFN-γ) and growth-regulated alpha protein (CXCL1). *P = 0.0388, **P = 0.0017. (**O**) Relative transcript levels measured in lung homogenates from Kras and Kras; Tfam^O/E^ mice showing the expression levels of IFN-related genes relative to controls (dotted line); *n* ≥ 4. Scatter plots show individual data points and the mean values ± SD. One-way ANOVA was used for the statistical analysis.

We next sought to understand whether the enhanced tumor burden of Kras; Tfam^O/E^ mice could be explained by changes in immune cell infiltration of lung tumors. Consequently, we prepared samples for flow cytometry analysis and designed a panel to quantify the proportion of several immune populations (fig. S2A). We found no significant differences in the overall proportion of CD45^+^ immune cells in the lungs of mice with different mtDNA levels (Fig. 3A), with specific cell populations similarly unchanged (Fig. 3, B to L). The frequency of myeloid cells—i.e., dendritic cells type 1 (cDC1), cDC2, alveolar macrophages, and interstitial macrophages (Fig. 3, B to E)—and lymphocytes—i.e., B cells, natural killer cells, CD4 T cells, regulatory T cells (T_regs_), and CD8 T cells (Fig. 3, H to L)—was comparable across the lungs of mice with different mtDNA levels. Quantification of granulocytes revealed that eosinophils were reduced in proportion in the Kras; Tfam^O/E^ model (Fig. 3G), whereas neutrophils were not altered (Fig. 3F). Overall, the immune cell populations infiltrating the lungs of tumor-bearing mice were markedly homogeneous in Kras and Kras; Tfam^O/E^ mice, suggesting that the observed differences in tumor burden were not caused by infiltrating immune cells.

mtDNA is known to have immunogenic properties and can cause an interferon response in tissue culture cells (*33*). To address whether this mechanism plays a role in our system, we measured the levels of 45 cytokines and chemokines in plasma from controls and Tfam^O/E^ mice with and without activation of KRAS^G12D^ expression in the lung (fig. S2, B to F). We found no significant differences in interferon-γ (IFN-γ) levels between tumor-bearing mice with different mtDNA levels (Fig. 3M and fig. S2F). These findings do not support the involvement of IFN-related pathways as a cause for the increased tumor burden observed in Kras; Tfam^O/E^ mice. In contrast, the growth-regulated alpha protein (CXCL1) levels in plasma were increased in Kras; Tfam^O/E^ mice (Fig. 3N and fig. S2D), which is in good agreement with previous reports that CXCL1 levels correlate with high tumor burden and poor prognosis in mouse models and patients with lung cancer (*34*–*36*).

We also investigated the expression of genes belonging to the IFN-signaling cascade by quantitative polymerase chain reaction (qPCR) in lung tissue homogenates (Fig. 3O). We found no significant differences in the expression of this set of genes when comparing lungs from Kras and Kras; Tfam^O/E^ mice. Overall, these data suggest that the enhanced tumor burden observed in Kras; Tfam^O/E^ mice is not due to major differences in the immune response.

### KRAS-driven lung tumors have increased mitochondrial capacity

**Fig. 4.**
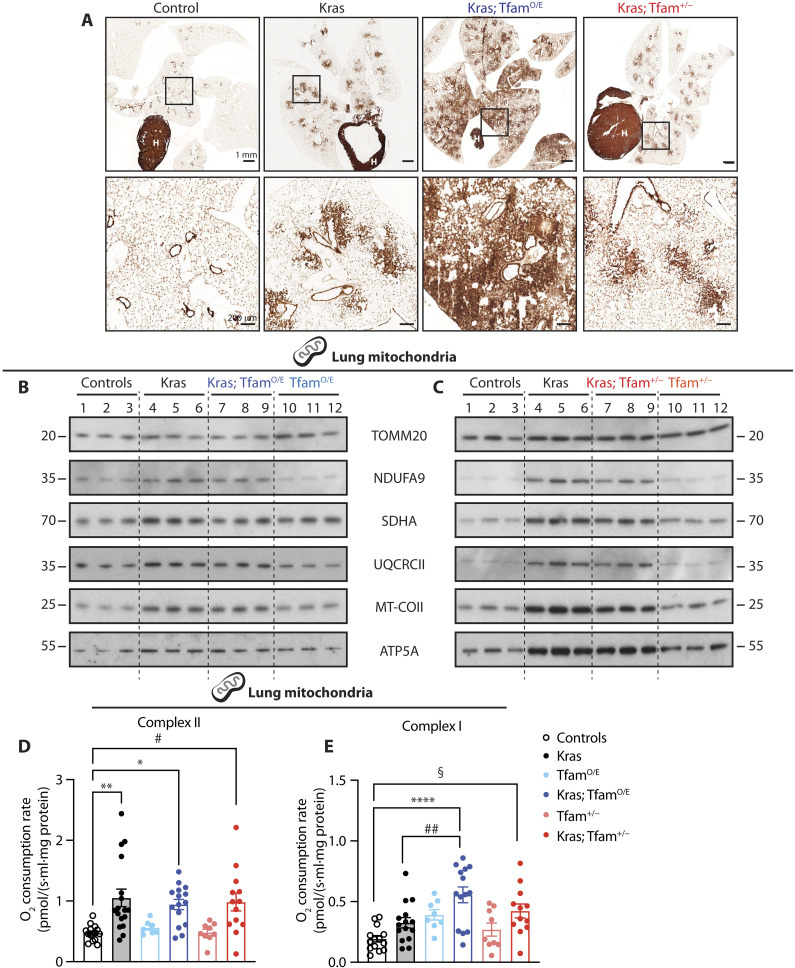
High mtDNA levels boost complex I–driven oxygen consumption rates. (**A**) COX enzyme activity performed on frozen lung tissue sections from controls, Kras, Kras; Tfam^O/E^, and Kras; Tfam^+/−^ mice. Scale bars, 1 mm (top) and 200 μm (bottom). H, heart. (**B** and **C**) Western blot analyses of the steady-state level of OXPHOS proteins in mitochondria isolated from lung tissue of controls, Kras, Tfam^O/E^, Kras; Tfam^O/E^ (B), Tfam^+/−^, and Kras; Tfam^+/−^ (C). Complex I: NADH dehydrogenase 1 alpha subunit 9 (NDUFA9), complex II: succinate dehydrogenase [ubiquinone] flavoprotein subunit A (SDHA), complex III: cytochrome *b-c1* complex subunit 2 (UQCRCII), complex IV: cytochrome *c* oxidase subunit 2 (MT-COII), and complex V: ATP synthase F1 subunit alpha (ATP5A). TOMM20 is used as loading reference. (**D** and **E**) Oxygen consumption rates through complex II (D) and complex I (E) measured by high-resolution respirometry in mitochondria isolated from lungs of controls, Kras, Tfam^O/E^, Kras; Tfam^O/E^, Tfam^+/−^, and Kras; Tfam^+/−^ mice. Scatter plots show individual data points ± SEM. Statistical significance was calculated with one-way ANOVA test. *P = 0.0229, **P = 0.0016, #*P* = 0.0148; §*P* = 0.0162, ##*P* = 0.0091, and ****P < 0.0001. Controls: *N* = 14; Kras: *N* = 15; Tfam^O/E^: *N* = 8; Kras; Tfam^O/E^: *N* = 14; Tfam^+/−^: *N* = 9; and Kras; Tfam^+/−^: *N* = 12.

We proceeded to investigate whether the increase in tumor burden of Kras; Tfam^O/E^ mice arose from a functional advantage linked to the OXPHOS capacity. To this end, we assessed mitochondrial function in lungs of wild-type, Tfam^O/E^, and Tfam^+/−^ mice with or without the activated oncogenic *Kras* allele. First, we qualitatively determined OXPHOS capacity in situ by performing histochemistry to assess complex IV (COX) enzyme activity on frozen lung tissue sections and found that normal lung tissue had low COX activity, as it is evident from the comparison with the high COX activity in the heart on the same tissue section (Fig. 4A). In contrast, KRAS-driven lung tumors had high COX activity in all mouse genotypes (Fig. 4A). These findings show that lung tumor tissue contains high OXPHOS activity, consistent with the observed increase of mitochondrial mass (Fig. 1, J and K). Furthermore, Western blot analyses of isolated lung mitochondria revealed increased steady-state levels of subunits belonging to all OXPHOS complexes when the expression of the mutant *Kras* allele was activated (Fig. 4, B and C). In contrast, no major differences in steady-state levels of OXPHOS proteins in the lung were observed when comparing wild-type mice with Tfam^O/E^ mice (Fig. 4B) or Tfam^+/−^ mice (Fig. 4C), consistent with our previous reports of unaltered OXPHOS capacity in other tissues of these mutant mice (*12*, *25*).

To investigate whether the increase of OXPHOS protein levels per mitochondrion had functional consequences, we performed high-resolution respirometry on isolated mitochondria from lung tissue of wild-type controls, Tfam^O/E^ mice, and Tfam^+/−^ mice with or without the mutant *Kras* allele. We observed higher respiratory chain capacity in mice carrying KRAS-induced tumors than in controls, Tfam^O/E^, or Tfam^+/−^ mice (Fig. 4, D and E), consistent with increased abundance of OXPHOS proteins in Kras mice (Fig. 4, B and C). Although respiration with substrates that enter electrons at the level of complex II was increased by the mutant *Kras* expression, different levels of mtDNA did not further impact respiration in lung mitochondria from the Kras; Tfam^O/E^ and Kras; Tfam^+/−^ mice (Fig. 4D). In contrast, respiration with substrates that enter electrons at the level of complex I was further increased in lung mitochondria when activation of mutant *Kras* expression was combined with increased mtDNA copy number (Fig. 4E). Our findings of a KRAS^G12D^-driven increase in respiration are consistent with a previous report showing in vivo activation of OXPHOS in mouse LUAD (*22*). Our data show that the increase in mtDNA levels in Kras; Tfam^O/E^ mice translates into greater respiration through complex I, which may provide a growth advantage and explain the increased tumor burden in these mice (Fig. 2, G and H).

### MtDNA levels directly affect tumor formation via an intrinsic mechanism

**Fig. 5.**
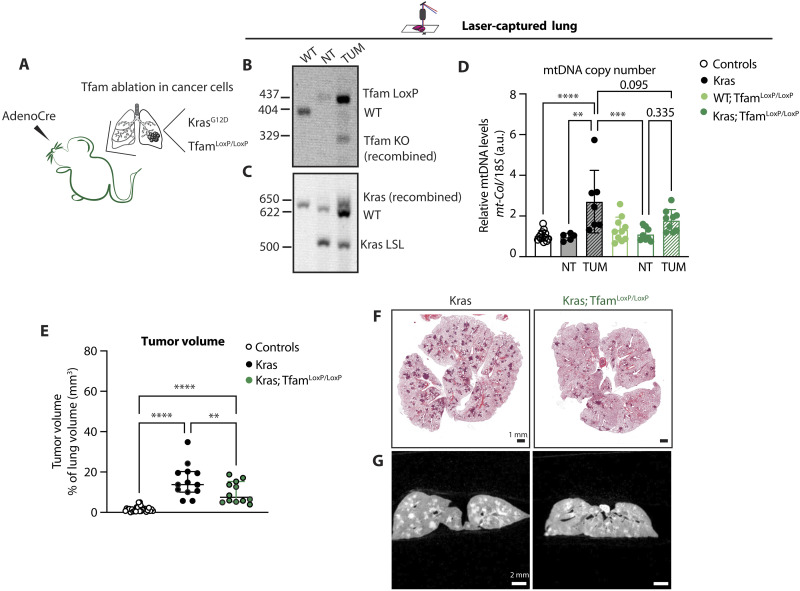
Depletion of mtDNA in tumor cells impairs tumor formation. (**A**) Schematics of the Kras; Tfam^LoxP/LoxP^ mouse model and expected outcome upon Cre recombination. (**B** and **C**) Recombination status of *Tfam* (B) and *Kras* (C) gene upon adenoCre induction in DNA extracted from laser-captured lung portions of controls [wild type (WT)] and Kras; Tfam^LoxP/LoxP^ mice. (**D**) Relative mtDNA levels normalized to nuclear DNA measured in laser-captured lungs portions from KRAS-induced tumor tissue (TUM) and normal surrounding tissue (NT) in controls (WT), Kras, Tfam^LoxP/LoxP^ (WT; Tfam^LoxP/LoxP^), and Kras; Tfam^LoxP/LoxP^ mice. Scatter plots show individual data points and the mean ± SD. Statistical significance is calculated with one-way ANOVA test. **P = 0.0010, ***P = 0.0005, and ****P < 0.0001. Controls: *N* = 12; Kras: *N* = 6; WT; Tfam^LoxP/LoxP^: *N* = 6; Kras; Tfam^LoxP/LoxP^: *N* = 8. (**E**) Quantification of tumor volume and airways in controls, Kras, and Kras; Tfam^LoxP/LoxP^ groups expressed as percentage of tumor over total lung tissue volume. Scatter plots show individual data points and the median + 95% CI for each group. Statistical significance is calculated with one-way ANOVA test. ****P < 0.0001 and **P = 0.0087. Controls: *N* = 27; Kras: *N* = 13; and Kras; Tfam^LoxP/LoxP^: *N* = 12. (**F** and **G**) Representative H&E-stained images of lung tissue sections (F) and μCT scans (G) of the lungs isolated from Kras and Tfam^LoxP/LoxP^ mice. Scale bars, H&E: 1 mm and μCT: 2 mm.

Our results show that the mtDNA levels are important for LUAD tumor burden, and we therefore proceeded to investigate the tumor cell–intrinsic role for mtDNA. To this end, we used a model that allows Cre-mediated *Tfam* ablation only in the tumor cells by creating Kras; Tfam^LoxP/LoxP^ mice (*12*). In this mouse strain, Cre-mediated activation of the mutant *Kras* allele will lead to simultaneous ablation of the *Tfam* gene (Fig. 5A), which will cause mtDNA depletion only in the cancer cells. We verified that Cre-mediated recombination of *Tfam* and *Kras* indeed occurred in the tumor cells by using PCR analysis of laser captured lung portions (Fig. 5, B and C, and fig. S2G) and determined mtDNA copy number (Fig. 5D). The Kras; Tfam^LoxP/LoxP^ mice showed a significant reduction of lung tumor burden when compared with Kras mice (Fig. 5, E to G). The small number of tumors (Fig. 5, E to G) and the absence of a significant increase in mtDNA copy number in lung tumors from Kras; Tfam^LoxP/LoxP^ mice (Fig. 5D) support our hypothesis of a cell-intrinsic essential role for mtDNA in tumor growth.

A previous study of cell lines and mice has suggested that KRAS-driven tumorigenesis is dependent on ROS production (*30*). The authors observed a similar reduction in lung tumor burden in Kras; Tfam^LoxP/LoxP^ mice as we do here, and, in addition, they identified a role for OXPHOS-driven ROS production in lung tumorigenesis. In our study, we did not detect any increase in superoxide-induced damage in lung mitochondria from Kras or Kras; Tfam^O/E^ mice when measuring the ACO2 enzyme activity (fig. S1H). Furthermore, we found no significant differences in ACO2 activity when lung mitochondria from control mice were compared with lung mitochondria from Tfam^LoxP/LoxP^, Kras, or Kras; Tfam^LoxP/LoxP^ mice all subjected to adenoCre inhalation (fig. S2H). These findings argue that mitochondrial superoxide damage may not be a major contributor of KRAS-driven tumorigenesis and that differences in cellular metabolic fluxes and OXPHOS capacities have an important influence on the tumor formation potential.

**Fig. 6.**
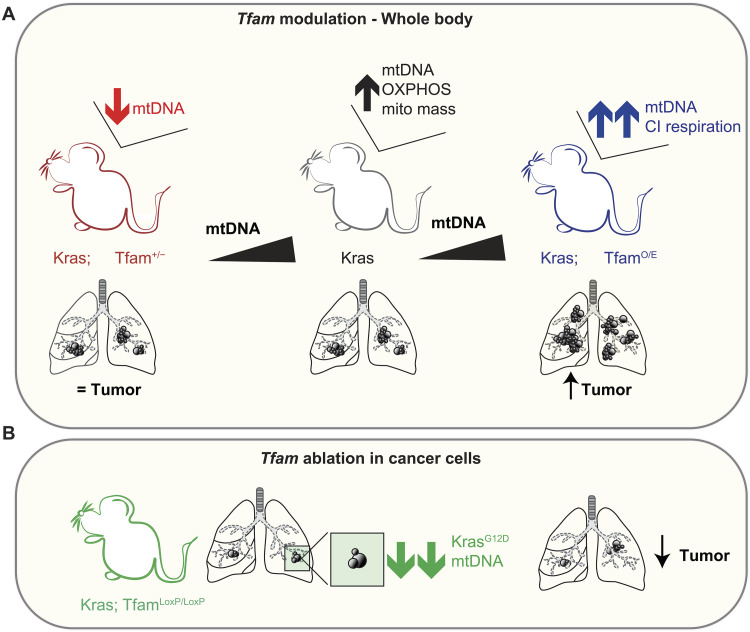
Summary of findings and proposed model. Schematic representation of the proposed model based on our findings. (**A**) Effect of whole body increased or reduced mtDNA levels on tumor burden. (**B**) Consequences of mtDNA depletion in tumor cells on tumor burden.

Together, the data presented here demonstrate that: (i) an increase in mtDNA copy number is necessary for KRAS-driven tumor formation in the lung (Fig. 6A); (ii) higher mtDNA content confers an advantage to tumor cells and results in increased tumor burden (Fig. 6A); and (iii) failure to increase mtDNA copy number in tumor cells impairs tumor growth (Fig. 6B). Our findings thus show that mtDNA levels directly influence lung cancer cell growth in vivo through cell-intrinsic mechanisms.

## DISCUSSION

We report a critical contribution of mtDNA to tumor progression in the lung. We identified a spontaneous increase in mtDNA levels, mitochondrial mass, OXPHOS proteins, and mitochondrial respiration driven by the activation of oncogenic KRAS^G12D^ expression. We also demonstrate the key importance of mtDNA levels by genetically altering mtDNA content in mice with lung cancer. We show that increasing mtDNA levels before activation of expression of mutant KRAS^G12D^ is sufficient to boost tumor burden, whereas mtDNA depletion in lung cancer cells reduces tumor burden in vivo. Analyses of large cancer datasets by us and others (*15*) show that a similar increase of both mtDNA levels and the expression of mitochondrial respiratory genes occurs in human LUAD. This strongly suggests that the mechanisms identified in our study are also relevant for human pathology. From a therapeutic point of view, targeting mitochondria for cancer treatment has raised a great interest, and many research groups, including ours, have developed compounds, e.g., the inhibitors of mitochondrial transcription, to block mitochondrial function as a mean to treat cancer (*37*–*44*). In this context, TFAM expression and/or mtDNA content could possibly be used as prognostic markers for lung cancer or to stratify patients that undergo mitochondria-targeted therapies in the future.

Previous reports have suggested that mitochondrial respiration might be especially important in LUAD, but not for other NSCLC types (*22*, *45*). Analyses of the Pan Cancer Analysis of Whole Genome (PCAWG) consortium database by Yuan *et al.* (*15*) identified elevated mtDNA levels in several common tumor types, including ovarian, colon, prostate, and breast cancers. We therefore speculate that the amount of mtDNA might also contribute to disease progression of other cancers. Therefore, experimentally assessing the in vivo consequences of mtDNA modulation in other tumor types would be an interesting question to address in the future.

We could not identify major differences in the immune cell populations infiltrating the lungs or cytokine levels in plasma when comparing Kras and Kras; Tfam^O/E^ mice. The findings argue that the increase in tumor burden did not arise from differences in immune cell infiltration or the immune response. However, it is possible that mtDNA copy number influences the activity of different immune cells, and it would be interesting to investigate whether the levels of mtDNA influence activation/exhaustion of certain immune cells, which could be pursued in a follow-up study.

Mechanistically, we propose that the high mtDNA levels might influence tumor growth by enhancing the respiratory flux through complex I. Complex I–driven respiration is essential to maintain the redox state of the cell, and previous work has reported that the regeneration of nicotinamide adenine dinucleotide (oxidized form) (NAD^+^) can be limiting for cancer cell growth (*7*, *45*). Our data suggest this may also apply to the Kras; Tfam^O/E^ lung cancer model, as we show that increased complex I–driven respiration correlates with increased tumor burden. Elevated complex I–driven oxygen consumption is likely to translate into higher oxidation rates of the reduced form of NAD^+^ (NADH), making more NAD^+^ available for the glycolytic flux leading to higher metabolic rates. It will be important to dissect this aspect further in future studies to pinpoint the exact molecular mechanism linking mtDNA content to the enhanced metabolic fluxes. Treatment of Kras; Tfam^O/E^ mice with specific complex I inhibitors or increasing the NAD^+^ pool in Kras mice could help to dissect these mechanisms further.

We observed that a strong depletion of mtDNA (Tfam^LoxP/LoxP^) in tumor cells is sufficient to slow down tumor progression in the lung. However, a mild decrease in mtDNA levels (Tfam^+/−^) neither impaired OXPHOS capacity nor decreased tumor burden, confirming the great buffering capacity of mitochondrial gene expression until a certain threshold is reached (*27*). These data are in accordance with what we observed previously in other tissues, showing that mild changes in mtDNA levels do not greatly affect mitochondrial function in normal conditions but may be important in pathology (*12*, *13*, *29*). KRAS-driven tumorigenesis has been reported to influence mitochondrial morphology and mitochondrial cristae structure (*45*–*47*), and there is an interesting interplay between mitochondrial dynamics and mtDNA replication (*48*). Further studies are required to shed light on the connection between mitochondrial structure, mtDNA replication, and tumor progression.

To conclude, we have identified a causative role for mtDNA in LUAD growth in vivo. High mtDNA content may prime tumor cells to have a higher mitochondrial potential conferring a functional advantage that ultimately results in increased tumor burden. This feature of lung cancer may be exploited as a vulnerability in future lung cancer therapy strategies.

## MATERIALS AND METHODS

### TCGA dataset analyses

Copy number estimates of LUAD mtDNA was obtained from (*14*) using only the whole-genome sequencing–based estimates. The values reported as “Log10-corrected tumor” was used as the measure for mtDNA copy number. LUAD expression data from TCGA was downloaded via the xenabrowser (https://xenabrowser.net/).The expression values, reported as the log 2(FPKM-UQ + 1) were used, where FPKM-UQ refers to the upper quartile normalized values of fragments per kilobase of transcript per million mapped reads, where the gene in the 75th percentile is used as a proxy for sequencing quantity. The dataset can be found at https://gdc-hub.s3.us-east-1.amazonaws.com/download/TCGA-LUAD.htseq_fpkm-uq.tsv.gz. Only tumor samples with both expression estimates and mtDNA copy number estimates were included for analysis, resulting in 107 samples. Genes included for regression analysis were those where at most 20 tumors had a log2(FPKM-UQ + 1) value below 0.1, totaling 23,857 genes. The cellular respiratory genes ID is GO:0045333.

### Mouse lines and tumor induction

Tfam^O/E^ (*25*, *28*), Tfam^+/−^, and Tfam^LoxP/LoxP^ (*12*) mice were previously generated in our laboratory. Kras^LSL-G12D^ mice were obtained from the Jackson Laboratories (RRID:IMSR_JAX:019104) (*23*, *24*). All mouse lines were backcrossed to C57BL/6N nuclear background. Animals were housed at 21°C in a 12-hour light/12-hour darkness cycle and fed ad lib. The project was approved by the Stockholm ethical committee (Stockholms djurförsöksetiska nämnd, ethical permit numbers 9646-2018, with amendment nos. 17140-2021, 19186-2019, and 12101-2023) and performed in accordance with the guidelines of the Federation of European Laboratory Animal Science Associations. Animals bearing wild-type or mutant *Kras* allele were treated at 6 to 7 weeks of age by delivering adenovirus encoding Cre recombinase (University of Iowa) through the nostrils, as previously described (*49*). After sedation with isoflurane (IsoFlo Vet, QN01AB06), 2.5 × 10^7^ plaque-forming units of AdenoCre dissolved in Minimum Essential Medium Eagle (Sigma-Aldrich, M4655) containing 10 mM CaCl_2_ were administered via nasal instillation. Mice were culled at approximately 6 months of age. Tumors were allowed to develop for 16 weeks, after which time, the mice were euthanized with CO_2_ followed by cervical dislocation, and the tissues were collected for analyses. Lungs were inflated with phosphate-buffered saline (PBS) through the trachea and collected for downstream applications. Freezing in liquid nitrogen, optimal cutting temperature (OCT) embedding or formaldehyde fixation (FA) was performed when needed for different downstream protocols. The study included both male and female mice in similar numbers. No significant differences in tumor burden between sexes were observed.

### Cytokines and chemokines quantification in plasma

After euthanasia by cervical dislocation, the blood was collected by cardiac puncture in anticoagulant-treated tubes (BD) and centrifugated at 2000*g* for 10 min at 4°C to isolate plasma. Plasma was then snap-frozen in liquid nitrogen and stored at −80°C until the measurement. Mouse cytokines/chemokines were quantified using the 45-Plex Discovery Assay Array (MD45) by Eve Technologies.

### Immune profiling in the lungs via flow cytometry

Mice were euthanized with CO_2_ and cervical dislocation, and the right atrium was flushed with PBS to eliminated blood from the vessels in the lungs. Lungs were also inflated through the trachea with PBS and collected, and single cells were obtained by using the Lung Dissociation Kit (130-095-927, Miltenyi Biotec) in a gentleMACS Octo Dissociator (Miltenyi Biotec). After red blood cell lysis was performed (Thermo Fisher Scientific, 00-4300-54), single cell suspensions were stained with the LIVE/DEAD staining (Thermo Fisher Scientific, L34967), and the antibodies are reported in Table 1. Flow Cytometry Staining buffer (fluorescence-activated cell sorting buffer, Thermo Fisher Scientific, 00-4222-26) was used for the washes and FoxP3 Staining buffer Set (Thermo Fisher Scientific, 00-5523-00) to permeabilize the cells and perform intracellular stainings.

**Table 1.** Antibodies used for flow cytometry. The samples were run on a BD LSR II SORP Flow Cytometer, and the data were analyzed using FloJo Software.

Antigen	Manufacturer	Catalog number
CD8	Thermo Fisher Scientific	56-0081-82
CD45	BioLegend	103107/08
Ly-6G	BioLegend	127651/52
CD11b	BD	563015
Siglec F	BioLegend	155507/8
CD19	BD	563235
CD3	BD	564010
CD4	BioLegend	100447
FoxP3	Thermo Fisher Scientific	61+5773+82
NK1.1	BioLegend	OATA00505-100
CD11c	BD	565451
F4/80	BD	565410

### Immunohistochemistry and H&E staining

At collection, lungs were inflated with PBS and 4% formaldehyde solution or with OCT compound (Tissue-Tek), depending on the downstream application. The tissue was either embedded in paraffin blocks or frozen in precooled methyl butane/isopentane (Sigma-Aldrich, M32631) and later stored at −80°C. The sections for histochemistry were placed on poly-l-lysine–coated slides (VWR), whereas sections used for laser capture microdissection were put on polyethylene naphthalate (PEN) membrane-framed slides (Leica Microsystems). Formalin-fixed paraffin-embedded lung sections were first deparaffinized in xylenes, rehydrated in descending alcohol gradient to water, and stained with Mayer’s hematoxylin solution (Merck). After bluing with 0.1% sodium bicarbonate, slides were washed and dehydrated with increasing concentrations of ethanol to xylenes, stained with eosin 0.25%, dehydrated, and mounted on coverslips slides. For laser capture microdissection experiments, the sections were stained with a similar protocol but air-dried before microcapture. COX staining was performed on 10-μm-thick OCT-embedded frozen sections as previously described (*29*). Lung sections were incubated with 0.2 ml of COX staining medium [500 μM cytochrome *c* (Sigma-Aldrich), adjusted at pH 7.0] and 0.8 ml of 5 mM 3,3 diaminobenzidine tetrahydrochloride [10 mg per tablet (Sigma-Aldrich) dissolved in deionized water, 0.2 M phosphate buffer at pH 7.0] mixed with 10 μl of catalase for 30 to 45 min at 37°C. After washes and dehydration, the slides were mounted on a coverslip and imaged.

### Laser capture microdissection

Hematoxylin and eosin (H&E)–stained PEN slides were cut in a Leica Microsystems LMD 7000 laser microdissection microscope as previously described (*29*). Tumor and normal lung portions were collected into single tubes containing 15 μl of DNA extraction buffer [50 mM tris-HCl (pH 8.5), 1 mM EDTA, and 0.5% Tween 20]. Tubes were incubated for 4 hours at 56°C and 10 min at 95°C. When paraffin-embedded, formalin-fixed sections were used in the laser capture procedure, tissue samples were collected into tubes containing lysis buffer from the QIAmp DNA micro purification kit (QIAGEN, 56304), and DNA was digested for 16 to 20 hours and extracted using the same kit (QIAGEN, 56304), according to the manufacturer’s specifications. Extracted DNA was frozen and used for downstream analyses.

### mtDNA copy number analyses

Total DNA extracted from whole tissue or laser-captured lung samples was used to measure mtDNA copy number via quantitative reverse transcription PCR (qRT-PCR). TaqMan probes against *Rnr1* or *mt-CoI* (*Rnr1*: Mm04260177_s1; *mt-CoI*: Mm04225243_g1, Thermo Fisher Scientific) were used and normalized against a probe against 18*S* nuclear DNA. The assays were performed in technical triplicates on 384-well reaction plates (Applied Biosystem) in final volumes of 10 μl according to the manufacturer’s guidelines.

### RNA isolation and transcript levels measurement

Total RNA from lung tissue was isolated using TRIzol reagent (Life Technologies, 15596018) and quantified by measuring the absorbance at 260 nm with a Nanodrop. RNA was retrotranscribed with the High-Capacity cDNA Reverse Transcription Kit (Applied Biosystem, 4368814), and the relative cDNAs were measured by qRT-PCR using TaqMan Universal Master Mix II and TaqMan probes (*Tfam*: Mm00447485_m1; *Ifng*: mm01168134_m1; *Irf3*: Mm00516784_m1; *Irf7*: Mm00516788_m1; *Stat1*: Mm00439518_m1; *Ifit1*: Mm00516788_m1; *Ifit44*: Mm00505670_m1; Thermo Fisher Scienitific) and normalized against *Actin B* cDNA. The assay was performed in technical triplicates in a 384-well plate format in 10-μl volume according to the manufacturer’s specifications.

### Mitochondria isolation from lung tissue

Mitochondrial isolation was performed by differential centrifugation as previously described (*29*). Lungs were homogenized in mitochondrial isolation buffer [320 mM sucrose, 1 mM EDTA, and 10 mM tris-HCl (pH 7.4), supplemented with 0.2% bovine serum albumin (Sigma-Aldrich)]. Subsequently, mitochondria were isolated from the homogenates by differential centrifugation (1000*g* and 4500*g*) in a swing rotor and stored at −80°C for further analyses.

### Protein extraction and Western blots analyses

For whole-tissue Western blotting analyses, snap-frozen lungs were pulverized in liquid nitrogen with mortar and pestle to obtain homogeneous tissue powders. Aliquots of whole-lung powders were homogenized in a homogenizer using tissue lysis buffer [25 mM Hepes, 5 mM MgCl_2_, 0.5 mM EDTA, 1X protease inhibitor, and 1% NP-40 (pH 7.5)] and centrifuged at 13,000*g* for 10 min at 4°C. The supernatant was collected, and protein concentration was measured. When mitochondria were used for Western blotting analyses, the isolation of mitochondria was followed by protein content quantification, and mitochondria were lysed by resuspension in water and 4x Laemmli buffer (NuPage, Thermo Fisher Scientific). A total of 20 μg of total proteins or 5 μg of isolated mitochondria was separated on NuPAGE gels (Life Technologies) depending on the separation profile needed. Proteins were transferred to polyvinylidene fluoride membranes or nitrocellulose membranes using iBlot system (Invitrogen) and blocked in 5% milk tris-buffered saline solution (TBS) containing 0.1% Tween for 1 hour. The membranes were incubated with primary antibodies overnight at 4°C. The following antibodies were used: vinculin (4650T, Cell Signaling Technology), HSC70 (sc-7298, Santa Cruz Biotechnology), TFAM (rabbit polyclonal antisera against TFAM generated using recombinant mouse protein), VDAC (ab14734, Abcam), HSP60 (AB1-SPA-807-E, Enzo Lifesciences), TOMM20 (ab78547, Abcam), NDUFA9 (ab14713, Abcam), SDHA (ab14715, Abcam), UQCRCII (ab14745, Abcam), MT-COII (rabbit polyclonal antisera against MT-COII generated using recombinant mouse protein), ATP5A (ab14748, Abcam), and ACO2 (#MA1-029, Thermo Fisher Scientific).

After washes in TBS-tween, the membranes were incubated with respective secondary antibodies (GE Healthcare) for 1 hour at room temperature. Proteins were detected using Clarity Western ECL Substrate (Bio-Rad).

### Tumor quantification

The quantification of tumor area was performed on TIFF images generated by scanning slides with H&E-stained sections. Images were segmented in Fiji (ImageJ) using a code for semi-automated quantification based on color threshold. The tumor areas (darker) were plotted as a function of total lung area for each image. Each data point is the average of two sections taken at different tissue depths. Quantifications were conducted blindly. For μCT imaging, lungs were fixed in 4% formaldehyde, dehydrated by ethanol gradient, and stained overnight with 1% iodine dissolved in absolute ethanol. The samples were washed once in ethanol 70% and scanned using a μCT (Quantum FX-CT, PerkinElmer, Waltham, MA, USA). The scans were performed under tube voltage of 90 kV, tube current of 200 μA, field of view at 20 mm, and scan time of 4.5 min. Quantum FX is a cone-beam μCT system, with the x-ray source and the detector rotated around a stationary sample by 360° during scanning. The three-dimensional CT data were reconstructed and analyzed using Analyze 12.0 (Biomedical Imaging Resource, Mayo Clinic, Rochester, MN). A semi-automatic segmentation was used to define total tumor volume and total lung volume based on x-ray attenuation difference between lung parenchyma and tumors. All quantifications were conducted blindly.

### High-resolution respirometry analysis of lung isolated mitochondria

Oxygen consumption was assessed in lung-isolated mitochondria using OROBOROS Oxygraph-2k high-resolution respirometer (OROBOROS O2K, Oroboros Instruments, Innsbruck, Austria). Mitochondria were isolated from total lung by homogenization in mitochondrial isolation buffer in 7-ml glass-glass Dounce homogenizer on ice. The homogenized tissue was then enriched for the mitochondrial fraction by differential centrifugation as described above. Mitochondrial protein content was assessed (Thermo Fischer Scientific, 23225) and total of 100 μg of mitochondrial protein was loaded into each OROBOROS O2K chamber at 37°C in Mir05 respiration buffer (0.5 mM EGTA, 3 mM MgCL_2_, 60 mM lactobionic acid, 20 mM taurine, 10 mM KH_2_PO_4_, 20 mM Hepes, and 110 mM d-sucrose). Oxygen consumption rates were continuously recorded throughout the experiment using the DatLab software (OROBOROS Instruments, Innsbruck, Austria). Complex I–linked respiration was determined by addition of 5 mM pyruvate, 0.5 mM malate, 10 mM glutamate, and 5 mM adenosine 5′-diphosphate (ADP). Complex II–linked respiration was determined by addition of 10 mM succinate and 5 mM ADP. Respiratory control ratio was calculated as described previously (*50*).

### ACO2 activity

Mitochondrial ACO activity was determined using the ACO activity assay kit (ab109712, Abcam) following the manufacturer’s recommendations. The assay was performed on isolated mitochondria from lungs. A total of 40 μg of frozen mitochondria was resuspended in ACO preservation solution (ab109712, Abcam). Each sample was analyzed in duplicates in a 96-well ultraviolet plate format. The conversion of isocitrate to *cis*-ACO was recorded by kinetic measurement at optical density at 240 nm for 30 min in the presence of isocitrate and manganese.

### Statistical analysis

Statistical analyses were performed with GraphPad Prism software using appropriate statistical tests detailed in each figure legend.
