# Supplementary material for: High mitochondrial DNA levels accelerate lung adenocarcinoma progression
Source: Sci Adv. 2024 Nov 1;10(44):eadp3481. doi: 10.1126/sciadv.adp3481 (PMC11529711; doi:10.1126/sciadv.adp3481)
Supplement: Supplementary file 1 — Figs. S1 and S2 Legends for movies S1 to S20 [file sciadv.adp3481_sm.pdf]

Science Advances
AAAS

Supplementary Materials for

High mitochondrial DNA levels accelerate lung adenocarcinoma progression

Mara Mennuni *et al.*

Corresponding author: Nils-Göran Larsson, nils-goran.larsson@ki.se

Sci. Adv. **10**, eadp3481 (2024)
DOI: 10.1126/sciadv.adp3481

The PDF file includes:

Figs. S1 and S2
Legends for movies S1 to S20

Other Supplementary Material for this manuscript includes the following:

Movies S1 to S20

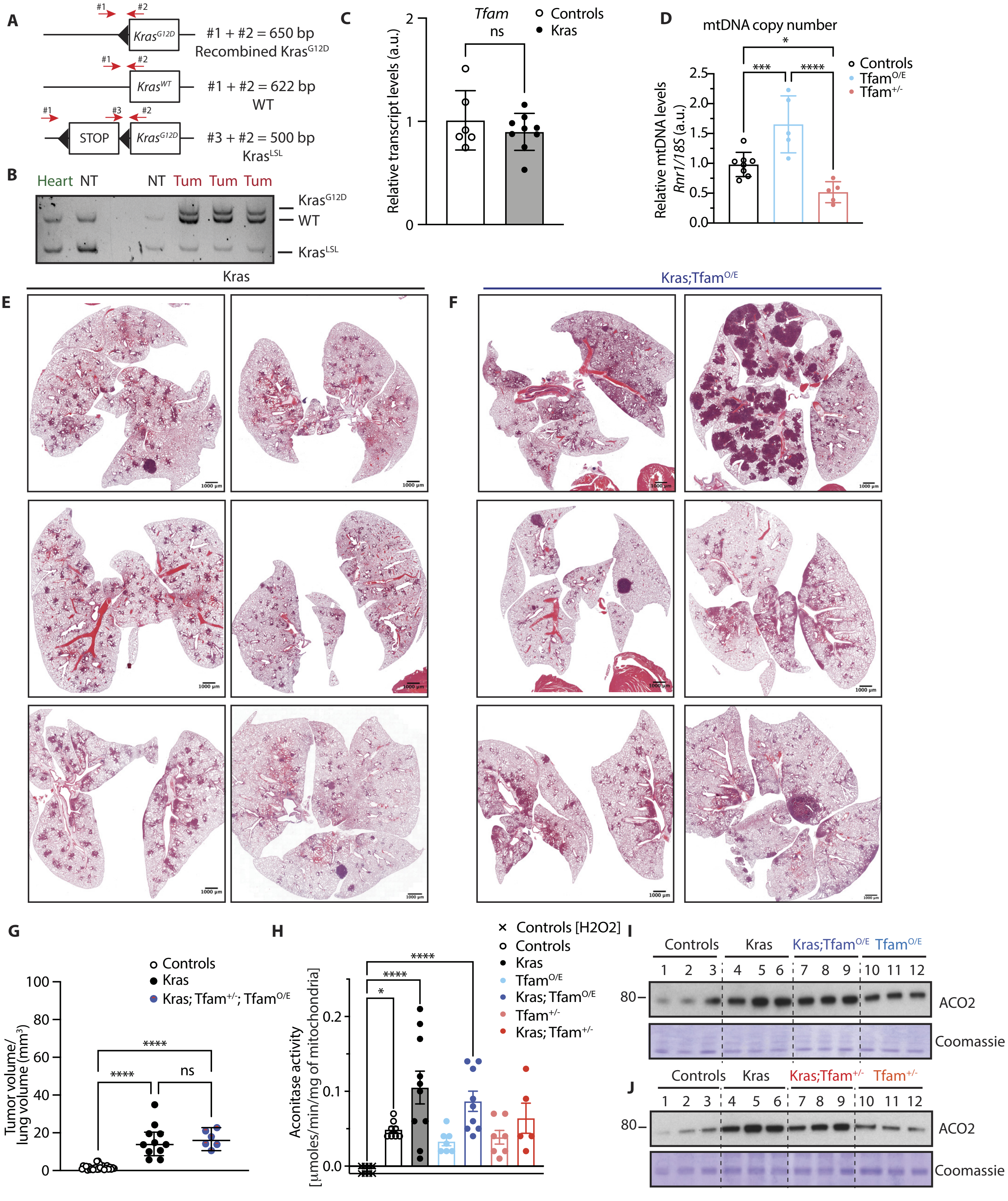

Figure S1. TFAM overexpression increases mtDNA copy number and enhances tumor burden in the lung without affecting superoxide-induced damage. **A**. Schematic representation of the expected PCR product sizes of different Kras alleles when using three primers in competition (23). **B.** Agarose gel separation of different Kras genetics variants in DNA extracted from laser captured tissue portions of Kras mice. Heart, normal lung tissue (NT) and tumor lesions (TUM). **C.** Relative *Tfam* transcript levels measured in whole lung homogenates collected from controls (wild-type) and Kras animals. Scatter plot columns report individual and the mean values ±SD. Data were normalized against Actin B expression and represent the ratio of controls. Two-tailed t-test was used for statistical analysis; controls: N =6; Kras: N = 9; ns = not significant ($P > 0.999$). **D**. Relative mtDNA levels normalized to nuclear DNA (*Rnr1/18S*) measured in lung tissue from the wild-type (controls), Tfam$^{O/E}$ and Tfam$^{+/-}$ animals. Scatter plots show individual data points and the mean values ± SD. One-way ANOVA was used for the statistical analysis; controls: N = 8, Tfam$^{O/E}$: N = 5, and Tfam$^{+/-}$: N = 5. *P = 0.0383; *** P = 0.0033 **** $P < 0.0001$. **E-F**. Representative images of H&E-stained lung tissue sections from Kras (E) and Kras; Tfam$^{O/E}$ (F) mice. Scale bars: 1mm. **G.** Quantification of lung tumor volume and airways in controls, Kras and Kras; Tfam$^{+/-}$; Tfam$^{O/E}$ mice expressed as percentage of tumor over total lung tissue volume. Scatter plots show individual data points and the median for each group. Statistical significance is calculated with one-way ANOVA test. ****$P < 0.0001$. Controls: N = 27; Kras: N = 11; Kras and Tfam$^{+/-}$; Tfam$^{O/E}$: N = 6. **H.** Aconitase enzymatic activity measured in mitochondria isolated from lungs as readout of superoxide induced damage. Scatter plot columns report individual data points and mean values ±SD. Statistical analysis was performed with standard one-way ANOVA test. All significant differences are reported on the graph. *P = 0.0306; ****$P < 0.0001$. Controls: N = 8; Kras: N = 10; Tfam$^{O/E}$: N = 8; Kras; Tfam$^{O/E}$: N = 9; Tfam$^{+/-}$: N = 7; Kras; Tfam$^{+/-}$: N = 5. Mitochondria from wild-type animals treated with H2O2 are used as negative control for the experiment. **I-J.** Representative western blots showing steady state levels of Aconitase 2 (ACO2) in mitochondria isolated from lung of controls (wild-type), Kras, Tfam$^{O/E}$, Kras; Tfam$^{O/E}$ (G), Tfam$^{+/-}$ and Kras; Tfam$^{+/-}$ (H). Coomassie staining is showed as loading reference.

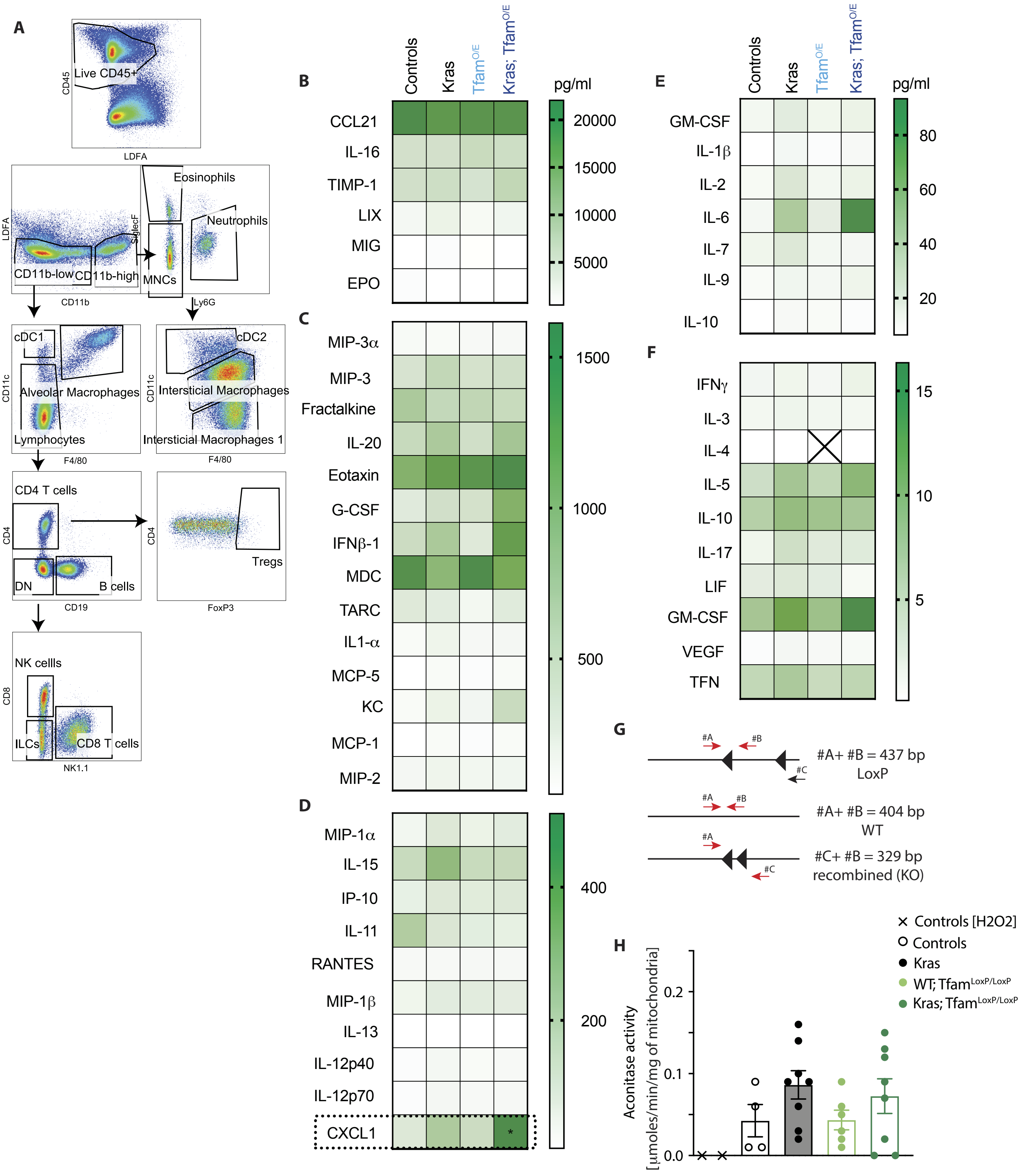

Figure S2. Cytokine levels in plasma are mostly unaffected by the activation of KRAS^{G12D} and by the increase in mtDNA levels. A. Gating strategy used for sorting single cells suspensions generated from mouse lung. Cell markers used are reported on the axes and specific immune populations in the graphs. **B-F.** Heatmaps showing the levels (pg/ml) of 45 inflammatory cytokines and chemokines measured in plasma samples collected from control (*N*=8), Kras (*N*=7), Tfam$^{O/E}$ (*N*=5), Kras; Tfam$^{O/E}$ (*N*=10) mice at 16 weeks after tumor induction. Heatmaps show the mean values of biological replicates. **G.** Schematic representation of the *Tfam* locus and expected PCR product sizes of different alleles (51) upon Cre recombination. **H**. Aconitase enzymatic activity measured in mitochondria isolated from lungs of Kras and Kras; Tfam$^{LoxP/LoxP}$ mice. Scatter plot columns report individual data points and mean values ±SD.

Supplementary auxiliary files:

Movie S1. Micro computed tomography stack of whole lung tissue from a WT animal (WT1).

Movie S2. Micro computed tomography stack of whole lung tissue from a WT animal (WT2).

Movie S3. Micro computed tomography stack of whole lung tissue from a WT animal (WT3).

Movie S4. Micro computed tomography stack of whole lung tissue from a WT animal (WT4).

Movie S5. Micro computed tomography stack of whole lung tissue from a Kras animal (Kras1).

Movie S6. Micro computed tomography stack of whole lung tissue from a Kras animal (Kras2).

Movie S7. Micro computed tomography stack of whole lung tissue from a Kras animal (Kras3).

Movie S8. Micro computed tomography stack of whole lung tissue from a Kras animal (Kras4).

Movie S9. Micro computed tomography stack of whole lung tissue from a Kras animal (Kras5).

Movie S10. Micro computed tomography stack of whole lung tissue from a Kras animal (Kras6).

Movie S11. Micro computed tomography stack of whole lung tissue from a Kras; TfamO/E animal (Kras; Tfam$^{O/E}$ 1).

Movie S12. Micro computed tomography stack of whole lung tissue from a Kras; TfamO/E animal (Kras; Tfam$^{O/E}$ 2).

Movie S13. Micro computed tomography stack of whole lung tissue from a Kras; TfamO/E animal (Kras; Tfam$^{O/E}$ 3).

Movie S14. Micro computed tomography stack of whole lung tissue from a Kras; TfamO/E animal (Kras; Tfam$^{O/E}$ 4).

Movie S15. Micro computed tomography stack of whole lung tissue from a Kras; TfamO/E animal (Kras; Tfam$^{O/E}$ 5).

Movie S16. Micro computed tomography stack of whole lung tissue from a Kras; TfamO/E animal (Kras; Tfam$^{O/E}$ 6).

Movie S17. Micro computed tomography stack of whole lung tissue from a TfamO/E animal (Tfam$^{O/E}$ 1).

Movie S18. Micro computed tomography stack of whole lung tissue from a TfamO/E animal (Tfam$^{O/E}$ 2).

Movie S19. Micro computed tomography stack of whole lung tissue from a TfamO/E animal (Tfam$^{O/E}$ 3).

Movie S20. Micro computed tomography stack of whole lung tissue from a TfamO/E animal (Tfam$^{O/E}$ 4).
